# Prophylactic retrievable inferior vena cava filters in spinal cord injured patients

**DOI:** 10.4103/2152-7806.72245

**Published:** 2010-10-30

**Authors:** Aaron Roberts, William F. Young

**Affiliations:** Indiana University School of Medicine, Fort Wayne Center, 2101 Coliseum blvd, USA; 1 Fort Wayne Neurological Center, 2622 Lake Ave, Fort Wayne, IN 468 05, USA

**Keywords:** Vena cava, spinal cord, filters, pulmonary embolus

## Abstract

**Background:**

Pulmonary embolus (PE) secondary to deep vein thrombosis (DVT) continues to be a major source of morbidity and mortality in trauma populations. Patients with cervical spinal cord injury (SCI) are particularly susceptible to developing this complication. Non-invasive methods of preventing SCI, such as lower extremity compression devices and anticoagulation, do not confer complete protection against DVT. Retrievable inferior vena cava filters (IVCFs) offer the advantage of both providing protection against PE and avoidance of long-term complications such as DVT, if removed in a timely fashion. Our goals in this study were to identify complications related to IVCF insertion and also to determine if prophylactic insertion of IVCF is effective in preventing PE in spinal cord injured patients.

**Methods:**

This was a retrospective single center study that involved cervical SCI patients who were admitted to Parkview Hospital, a level II trauma center, from January 2003 to December 2009 and underwent placement of a prophylactic IVCF within 72 hours of admission. Patients were identified from a prospectively maintained trauma registry.

**Results:**

During a 6-year period, 45 spinal cord injured patients were identified, who underwent placement of a prophylactic IVCF. There were 37 men and 8 women. There were no short-term complications associated with peripheral intravenous catheter (PIVC) insertion. Seventeen of the 45 (37%) patients underwent successful removal of the filter within 6–8 weeks of insertion. Twenty patients did not return for removal during the 6–8 week period for removal and eight patients were lost to follow-up. None of the patients who underwent prophylactic IVCF placement sustained a PE.

**Conclusion:**

Our results suggest that the use of retrievable prophylactic IVCF is a safe procedure and has the added benefit of preventing the long-term lower extremity thrombotic complications associated with their use. Even though none of the patients sustained a PE, definitive conclusions regarding the efficacy of IVCF in preventing PE could not be made due to the small sample size of our study.

## INTRODUCTION

Previous studies have shown that the use of prophylactic inferior vena cava filters (IVCFs) significantly reduces the rate of pulmonary embolus (PE) and deep vein thrombosis (DVT) when compared to other trauma patients; however, their use remains controversial.[1,3,5–10] Permanent IVCFs are associated in the long term with caval thrombosis leading to debilitating lower extremity venous stasis disease. Retrievable have the potential benefit of preventing this complication through early removal. In 2003, we began a program of implanting prophylactic IVCF in high-risk cervical spinal cord injured patients in order to prevent pulmonary embolism. In this study, we analyzed our experience with prophylactic IVCF in patients who sustained cervical spinal cord injury (SCI) and were not felt to be candidates for early use of Low Dose Unfractionated Heparin (LDH) or Low Molecular Weight Heparin (LMH).

## MATERIALS AND METHODS

This was a retrospective, single-center review of cervical spinal cord injured patients admitted to Parkview Hospital, who underwent placement of prophylactic IVCF. Parkview Hospital is a 573-bed institution in Fort Wayne, Indiana. It is a level II trauma center verified by the American College of Surgeons. The catchment area for the hospital includes more than 35 surrounding counties, including some in the neighboring states of Ohio and Michigan. The trauma center serves an estimated population of approximately 2.4 million people in this mixed urban and rural area. An institutional review board approved this project before beginning the research.

As a part of the verification of its trauma program, Parkview hospital maintains a prospective database of all trauma patients evaluated through the program. This database was queried retrospectively to identify all cervical spinal cord injured patients who underwent placement of an IVCF. These patients were then reviewed in order to identify those who had prophylactic IVCF placed. Demographic data were collected on all patients, including age, sex and Injury Severity Score (ISS). Criteria for placement of a prophylactic IVCF included severe cervical SCI resulting in quadriplegia or quadriparesis, and relative contraindications for the use of LDH or LMH (e.g., need for spinal surgery stabilization, concomitant injuries such as cranial trauma). Filters were placed within 72 hours of admission. They were all placed by an interventional radiologist in the radiology suite. Before filter placement, an inferior vena cavagram was performed to evaluate the inferior vena cava diameter, assess renal veins and to exclude the presence of thrombus within the cava, which would prevent placement of a filter. Retrievable titanium filters were placed in all patients. All patients were discharged to a rehabilitation center or extended care facility after leaving the hospital. Follow-up visits were conducted by the senior author.

## RESULTS

During the period 2003–2009, 11,642 patients were evaluated by the trauma service and included in the database. From the prospectively maintained trauma registry, 45 patients were then identified, who sustained traumatic cervical SCI and had a prophylactic IVCF placed within 72 hours of admission to the intensive care unit. In addition, data were obtained from the senior author’s practice database. All inpatient and outpatient medical records were reviewed. There were 37 men and 8 women. The age range was 17–67 years. The mean age was 39.7 years. The mean ISS score was 34.2. 100% of the patients in the study had an ISS score greater than 20. Thirty-six patients underwent cervical spine surgery for stabilization and nine patients underwent placement of a halo/vest for stabilization of the fracture. The six patients who underwent halo/vest placement had concomitant injuries which made the use of early prophylactic anticoagulation, contraindicated.

Filters were placed by an interventional radiologist in the radiology department. There were no complications related to IVCF insertion. Patients were placed on prophylactic anticoagulant therapy 1 week after injury (subcutaneous lovenox or heparin). There were no deaths during the follow-up period. Thirty-seven were available for long-term follow-up (6–12 months post-insertion). Seventeen of the 45 (37%) IVCF placements were removed successfully 6–8 weeks after insertion. Twenty patients did not return during the designated time for removal (6–8 weeks). The interventional radiology physicians who placed the IVCF felt that attempts at removal after this time period may increase the incidence of potential complications associated with retrieval (e.g., inferior vena cava injury). None of the patients who underwent placement of a filter sustained a PE during the study period. One patient who sustained a severe SCI was deemed ineligible for placement of a filter sustained a PE 3 weeks after injury. This patient had a traumatic retroperitoneal hematoma and it was felt that placement of a filter would be problematic. This patient was placed on therapeutic anticoagulation and ultimately had an excellent outcome.

## DISCUSSION

IVCFs were first utilized in the 1960s for the prevention of PE. The routine use of prophylactic IVCF has been questioned due to concerns regarding acute complications related to filter insertion (perforation) and late complications relating to filter migration and caval thrombosis. The incidence of acute complications with IVCF insertion ranges from 0 to 9.2%.[2]

The most common long-term complication due to IVCF is lower extremity venous stasis which can occur in more than 40% of patients with permanent filters.[4] Patients with IVCFs have a much higher incidence of DVT when compared to those patients receiving only anticoagulation (20.8% vs. 11.6%). Additional long-term complications associated with IVCFs include fracturing of the filter struts, and perforation of the vena cava and adjacent viscera (aorta, liver, small intestines, and spinal column). IVCFs have a reported insertion related mortality rate of 0–0.5%. Moreover, they are not 100% effective against PE.[7]

A major criticism of prophylactic IVCF insertion is its permanency which increases the risk of caval thrombosis and migration.[12] This criticism has become less of an issue due to the advent of titanium retrievable filters utilizing endovascular techniques. In theory, these devices would provide high-risk patients with greater protection from PE while avoiding the long-term complications of a permanent filter.[6] However, rates of actual removal have been low ranging from as little as 18% up to 32% in previous studies.[4]

Previous investigators have also documented their experience with the placement of prophylactic IVCFs in patients who sustained a SCI.

We identified a total of 215 SCI patients in 10 studies, who had undergone prophylactic IVCF placement.[1,3,5–7,9–11,13] All of the studies were part of the relatively recent medical literature (1994–2006). All studies were retrospective. The mean sample size from the studies was 21.5. The range for sample size was 4–47. The incidence of DVT could not be determined since many studies did not include this data separately for SCI patients. One of the 215 patients who underwent prophylactic IVCF placement developed PE in the studies reviewed (0.46%). Complications from prophylactic IVCF placement in the 215 patients included filter migration, caval perforation and caval thrombosis. The overall complication rate could not be determined since most studies included all types of trauma patients and did not separate out the complication rate for SCI patients. There were no deaths directly attributable to IVCF.

The total cost of 10 weeks of LMH therapy and 2 weeks of Intermittent Pneumatic Compression Devices IPCs is $ 3,392.68.[3] The cost of placing an IVCF ranges from $57,377 to $123,682. If there are an estimated 11,000 new SCI patients annually in the US, (IVCF’s) could potentially add approximately $500 million to medical expenditures. There are ways to reduce the cost of filters, such as performing the insertion in the intensive care unit as opposed to a radiology suite or an operating room.[3,11] However, cost remains an important factor in any risk versus benefit analysis of their use.

## CONCLUSION

As hospital complications become increasingly classified as “never events”, it will necessarily place an increasing burden upon health care providers to “prevent events”. This is particularly true for those who care for high-risk patients such as those sustaining SCIs. Health care policy makers have sometimes not appreciated the differences among patient sub-groups in terms of risk of adverse events.[2]

Our study confirms previous reports that demonstrate the efficacy of prophylactic IVCF in preventing venous thromboembolism in SCI patients. Given the devastating consequences that can result from PE, we advocate the use of prophylactic IVCF in SCI patients who cannot undergo early prophylactic anticoagulation, such as those who have a major head injury and those undergoing early spinal reconstructive surgery. Moreover, with the use of retrievable filters, problems with caval thrombosis may be decreased, since patients can be placed on long-term anticoagulation once it is deemed safe and the filter can be removed.

Current studies including our own are limited by the fact that they are retrospective and include a small number of patients. Ultimately, a prospective, randomized, multicenter trial may be required before more definitive recommendations can be made. However, such a study would have to include thousands of patients and also involve autopsy confirmation that the cause of death was due to PE. The expense of such a study may preclude its development. Therefore, aggregate retrospective analysis may be the only practical means of providing recommendations. In conclusion, given the known increased incidence of DVT and the devastating consequences of PE in this population, it seems reasonable to place retrievable prophylactic IVCFs in selected SCI patients, given the low complication rate associated with their usage.
